# GeneSCF: a real-time based functional enrichment tool with support for multiple organisms

**DOI:** 10.1186/s12859-016-1250-z

**Published:** 2016-09-13

**Authors:** Santhilal Subhash, Chandrasekhar Kanduri

**Affiliations:** Department of Medical Genetics, Institute of Biomedicine, The Sahlgrenska Academy, University of Gothenburg, Gothenburg, SE-40530 Sweden

**Keywords:** Gene enrichment tool, Real-time analysis, KEGG, Gene Ontology, Cancer enrichment, Pathway enrichments, Functional enrichments

## Abstract

**Background:**

High-throughput technologies such as ChIP-sequencing, RNA-sequencing, DNA sequencing and quantitative metabolomics generate a huge volume of data. Researchers often rely on functional enrichment tools to interpret the biological significance of the affected genes from these high-throughput studies. However, currently available functional enrichment tools need to be updated frequently to adapt to new entries from the functional database repositories. Hence there is a need for a simplified tool that can perform functional enrichment analysis by using updated information directly from the source databases such as KEGG, Reactome or Gene Ontology etc.

**Results:**

In this study, we focused on designing a command-line tool called GeneSCF (Gene Set Clustering based on Functional annotations), that can predict the functionally relevant biological information for a set of genes in a real-time updated manner. It is designed to handle information from more than 4000 organisms from freely available prominent functional databases like KEGG, Reactome and Gene Ontology. We successfully employed our tool on two of published datasets to predict the biologically relevant functional information. The core features of this tool were tested on Linux machines without the need for installation of more dependencies.

**Conclusions:**

GeneSCF is more reliable compared to other enrichment tools because of its ability to use reference functional databases in real-time to perform enrichment analysis. It is an easy-to-integrate tool with other pipelines available for downstream analysis of high-throughput data. More importantly, GeneSCF can run multiple gene lists simultaneously on different organisms thereby saving time for the users. Since the tool is designed to be ready-to-use, there is no need for any complex compilation and installation procedures.

**Electronic supplementary material:**

The online version of this article (doi:10.1186/s12859-016-1250-z) contains supplementary material, which is available to authorized users.

## Background

**Fig. 1:**
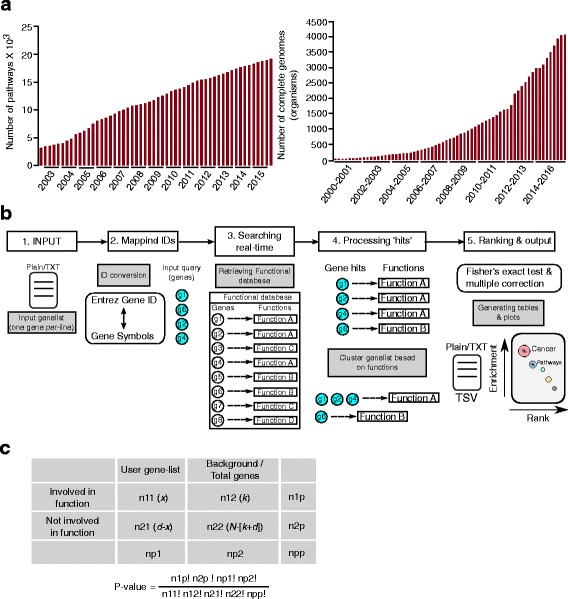
Summary of methodology used in GeneSCF. **a** The bar graphs shows the increase in information (yearly) stored on KEGG database such as number of genes covered (*left panel*), number of supported organisms (*right panel*) and number of functional pathways (*bottom-left panel*). **b** The workflow starts with the input gene list from the user in the form of gene symbols or Entrez GeneID’s as plain text format and searches the remote database based on the preferred choice of database. The gene hits in the database are clustered based on matching functions and followed by ranking of enriched functions using different statistical methods. The final output from GeneSCF will be plain text and tab-separated list of enriched functions and an optional enrichment plot. **c** The contingency table represents the matrix to perform Fisher’s exact test for calculating significance level (*P*-values). The equation below the table is used by GeneSCF to calculate *P*-values. The contingency table is explained in detail in article sub-section Enrichment analysis under Implementation and usage main section

Functional interpretation of large-scale data from high-throughput sequencing and microarray experiments is a crucial part of biological research to gain mechanistic insights of affected genes in a particular biological context. To this end, researchers often use a huge volume of functional information stored and organized in different databases like KEGG [1], Gene Ontology (GO) [2] and Reactome [3]. These dedicated databases, which provide gene level functional information collected from previous studies, are used as a source for the majority of the functional enrichment tools. The functional information in the databases used by the enrichment analysis tools is increasing drastically and it is difficult for those tools to incorporate the updated information frequently (Fig. 1a). Therefore there is a need for an automated functional enrichment tool that can directly use functional information in real-time from the source databases such as KEGG, Reactome or Gene Ontology (GO) to perform enrichment analysis. Keeping in mind the problems of using updated databases associated with currently available functional enrichment tools, we have designed a Unix based command line tool called GeneSCF (Gene Set Clustering based on Functional annotations). GeneSCF accepts a simple gene list as an input and searches the genes in user-preferred databases in a real-time-up-to-date manner and ranks them using standard statistical methods (Fig. 1b).

## Implementation and usage

GeneSCF uses PERL modules to query input genes and ranks the enriched functions using the overlapping gene hits. It can provide enrichment results using optional bubble plots that require R package and ggplot2 to be pre-installed in the system. The modules for updating the databases were also written using PERL and bash scripting with basic Unix commands. GeneSCF is an installation and compilation free tool, which runs on pre-installed programs from Linux such as PERL and with some basic Unix commands. All the features of this tool have been extensively tested on prominent Linux distributions within bioinformatics communities such as, Ubuntu, CentOS and Mint.

### General usage

GeneSCF is available for download from project homepage http://genescf.kandurilab.org as a compressed file and can be simply executed by uncompressing the file without any need for special installation procedures. The following is the template for GeneSCF command-line usage.

>> ./geneSCF --mode=[update|normal] --infile=[input_list] --gtype=[gid|sym] --output=[outpath/] --database=[reference_database] --plot=[yes|no] --background=[TotalGenes] --organism=[organism_codes]

GeneSCF supports two different modes, *update* and *normal* mode (--*mode* parameter) while performing enrichment analysis. In *update* mode, this tool retrieves the functional information from corresponding database and organism in real-time and performs the enrichment analysis. On the other hand, in *normal* mode the enrichment analysis will be performed on previously retrieved functional databases using *update* mode. The GeneSCF tool download comes with default database for *Homo sapiens*.

### GeneSCF input

The standard input for GeneSCF is plain text document (text/plain) containing a list of genes in the form of official gene symbols (Example, HGNC for human, --*gtype*=sym) or Entrez GeneID [4] (--*gtype*=gid) (Fig. 1b). The input gene list by definition contains genes that are predicted to be affected in any particular biological study. For example, genes with abnormal gene expression patterns or abnormal epigenetic modifications can be used as the input.

### Reference databases in GeneSCF

In biological research, selection of the appropriate database is an important step in predicting relevant functional information for the performed experiments. GeneSCF provides this option in a very specific and flexible manner by using resources from KEGG, GO, Reactome and NCG [5]. KEGG and Reactome cover information on different molecular pathways involved in metabolism, gene regulation, cellular processes, disease and signaling pathways. The traditional GO database is a collection of three different functional categories, Biological Process (GO_BP), Molecular Function (GO_MF) and Cellular Component (GO_CC). Cellular Component covers information on the location of genes in different sub-cellular and macromolecular complexes. NCG is a cancer specific database contains 2000 genes implicated in 66 cancer types. GeneSCF supports approximately 4000 species from KEGG (Additional file 1), 36 species from GO [6] (Additional file 2). On the other hand, the Reactome and NCG databases support only *Homo sapiens*. Multi-organism supported databases KEGG and GO can be selected by using the organism codes provided with the GeneSCF tool using the --*organism* parameter (Additional files 1 and 2). One of the important features of GeneSCF is that it can easily retrieve all the newly added organisms from the databases by using the appropriate organism code from corresponding database. Hence there is no restriction for number of supported organisms when using GeneSCF.

### Preparing multiple reference databases prior to enrichment run

Most of the functional enrichment tools require the reference functional database to be prepared and formatted by users following the guidelines of corresponding enrichment tools. In this context, GeneSCF overcomes these problems by using an integrated module, called ‘*prepare_database*’, to extract the updated functional information from any number of multi-organism supported databases by mentioning the name of the database with the organism code. This module can be executed by the simple command.

>> ./prepare_database -db=[GO|KEGG|REACTOME] -org=[organism_code]

The above command retrieves functional information from corresponding database using HTTP (Hypertext Transfer Protocol) service. The information will be extracted on the basis of user query for selected database and organism. For KEGG, the pathways and the genes can be retrieved using KEGG rest API, http://rest.kegg.jp/list/pathway/ORGANISM_CODE and http://rest.kegg.jp/link/genes/PATHWAY, and for Gene Ontology using HTTP service http://purl.obolibrary.org/obo/go.obo and http://geneontology.org/gene-associations/gene_association.ORGANISM_CODE.gz. For REACTOME the current file can be accessed from http://www.reactome.org/download/current/ReactomePathways.gmt.zip.

### Enrichment analysis

GeneSCF searches the input gene list against a user preferred database and retrieves the gene hits along with the corresponding functions. Obtained gene hits are clustered based on the functional information extracted from the database and each cluster is further ranked based on the statistical significance (Fig. 1b). The rankings are based on statistical tests performed on the values from contingency table. The contingency table in Fig. 1c represents,

n11 | x : User supplied genes involved in specific biological function

n21 | d-x : User supplied genes NOT involved in specific biological function

n12 | k : Total genes in specific biological function

n22 | N - [ k + d ] : All genes from the genome

whereas,

d : total deregulated genes (User supplied gene list)

x : User supplied genes involved in specific biological function

k : genes involved in specific biological function

N : total genes in background

From the contingency table, GeneSCF calculates the significance of enrichment using Fisher’s Exact test [7] with standard multiple testing correction methods by utilizing available PERL statistical modules. The multiple hypothesis correction methods includes Benjamini and Hochberg, Hommel singlewise process, Bonferroni single-step process, Hochberg step-up process and Benjamini and Yekutieli multiple hypothesis correction factors. The PERL modules used for achieving statistics is, Fisher’s Exact test, *Text::NSP::Measures::2D::Fisher* and Multiple testing correction (FDR or *q*-value), *Statistics::Multtest*.

### GeneSCF output

The final output from GeneSCF is a table (text/tab-separated file, TSV) with list of ranked functions based on the number of hits from the user provided gene list. It also contains column with probability value (*P*-value) obtained by Fisher’s exact test using the contingency table (Fig. 1c) and also columns containing False Discovery Rate (FDR) values using different multiple hypothesis correction methods.

**Fig. 2:**
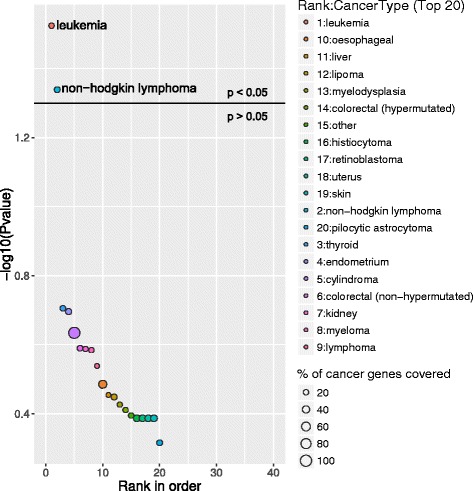
Enrichment of CLL-associated DEGs in different cancer types. Bubble plot represents the top 20 enriched cancer types in CLL DEGs using NCG as a reference database. The X-axis represents the ranks of cancer type based on *P*-values and Y-axis represents log-transformed *P*-values; the size of each bubble in the plot represents the percentage of genes covered in the corresponding cancer type by CLL associated DEGs. The enriched relevant cancer type to the study is highlighted in the plot and in this case study leukemia and non-hodgkin lymphoma are highlighted. The horizontal line in the plot represents above and below significance level of *p* < 0.05

There is also an optional graphical output by including the --*plot* parameter in GeneSCF tool. If the plot option is enabled, GeneSCF provides the top 20 enriched functions predicted from the given input gene list as a graphical plot. For this purpose GeneSCF uses a function from the R package called ‘ggplot2’ [8]. This is an optional dependency to visualize the output as bubble plot with the top 20 functions ranked based on their *P*-values predicted by GeneSCF (example see Fig. 2).

## Results and discussion

The methodology used in GeneSCF has been already implemented in Mondal et.al, 2015 [9] using GeneSCF v1.0 (supports only human database and no update mode) for finding *MEG3* and *EZH2* regulated pathways using microarray and RNA sequencing (RNA-seq) techniques. Most of the affected pathways predicted by GeneSCF were consistent between the deregulated genes from the microarray and RNA-seq experiments. The functional role of genes from well-studied pathways like TGF-β signaling was revalidated based on the predictions from GeneSCF. To further validate the performance and prediction level, the GeneSCF v1.1 was tested on previously published datasets from two studies based on different techniques. In the first study, we have used transcriptome sequencing data from chronic lymphocytic leukemia (CLL) patients and healthy individuals, and in the second study, chromatin immunoprecipitation (ChIP) sequencing data to identify p53 bound regions on genome-wide scale (Additional file 3). Later GeneSCF v1.1 was also compared with different enrichment tools to show the importance of updated information in performing enrichment analysis. The enrichment analysis by GeneSCF on *update* or *normal* mode in this study was performed using KEGG release 77.1 and Gene Ontology release 3/16/2016.

### Case study 1: CLL deregulated genes were predicted to be enriched in the cancer type leukemia

**Fig. 3:**
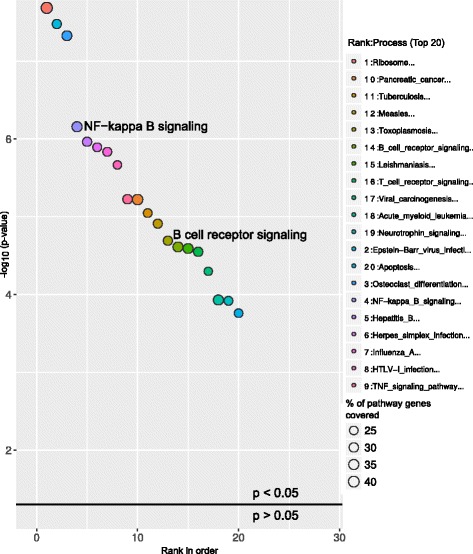
Pathway enrichment of CLL-associated DEGs using KEGG. Bubble plot represents the top 20 enriched pathways in CLL DEGs using KEGG as a reference database. The X-axis represent the ranks of pathways based on *P*-values and Y-axis represents log-transformed *P*-values; the size of each bubble in the plot represents the percentage of genes covered in corresponding pathways by CLL associated DEGs. The enriched pathways relevant to the study are highlighted in the plot and in this case study NF-kappa B and B cell receptor signaling pathways are highlighted. The horizontal line in the plot represents above and below significance level of *p* < 0.05

The study from Ferreira et.al, 2014 [10] identified several differentially expressed genes (DEGs) between CLL patients and healthy individuals. To test the reliability of GeneSCF, we used these CLL DEGs as input with the reference database as NCG, containing information on cancer genes. GeneSCF predicted the DEGs as leukemia and lymphoma specific genes enriched with an error rate of less than 5 % and false discovery rate within 10 % (Fig. 2, Additional file 4). The pathway enrichment of CLL associated DEGs using KEGG database predicted well known CLL deregulated pathways such as B cell receptor [11] and NF-kapp B signaling [12] within the top 20 enriched pathways (Fig. 3, Additional file 5).

### Case study 2: pathway analysis using genes from ChIP sequencing dataset

**Fig. 4:**
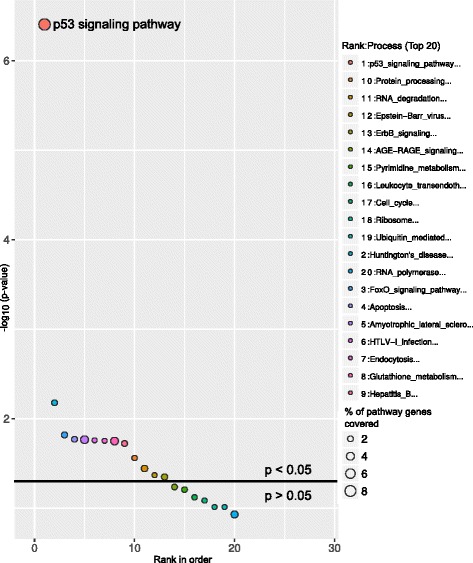
Molecular pathways deregulated by p53 bound genes at the 0 h time-point. Bubble plot shows the enriched p53 signaling pathway by p53 protein bound genes in HCT116 cell line treated with DNA-damage-inducing drug at the 0 h time point. The X-axis represent the ranks of pathways based on *P*-values and Y-axis represents log-transformed *P*-values; size of each bubble in the plot represents the percentage of genes covered in corresponding pathways. The horizontal line in the plot represents above and below significance level of *p* < 0.05

**Fig. 5:**
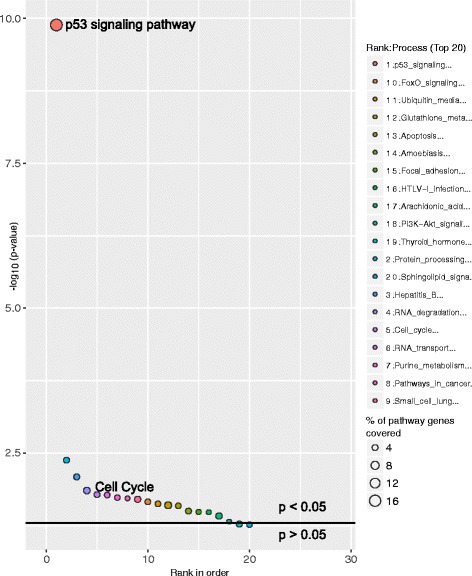
Molecular pathways deregulated by p53 bound genes at the 12 h time-point. Bubble plot shows the enrichment of p53 signaling pathway and Cell cycle process by p53 protein bound genes in HCT116 cell line treated with DNA-damage-inducing drug at the 12 h time point. The X-axis represent the ranks of pathways based on *P*-values and Y-axis represents log-transformed *P*-values; the size of each bubble in the plot represents the percentage of genes covered in corresponding pathways. The horizontal line in the plot represents above and below significance level of *p* < 0.05

ChIP is a well-known technique to identify binding sites for DNA binding transcription factors across the whole genome. A recent study by Sánchez Y et al, 2014 [13] performed ChIP-seq on a well-known transcription factor p53 tumor suppressor protein and found several p53 bound genes across the whole genome. This ChIP experiment was performed in a HCT116 cell line treated with a DNA-damage-inducing drug at different time points. In our study we used the p53 bound genes from 0 to 12 h as two different sets and performed the pathway analysis using GeneSCF (0 h, Fig. 4 and; 12 h, Fig. 5). p53 signaling was ranked as the most significantly affected pathway from the p53 bound genes at both 0 and 12 h time points. The consistency of the predicted results was maintained when using both KEGG (Figs 4 and 5, Additional files 6 and 7) and GO databases (Additional files 8 and 9). At the 12 h time point, in addition to the p53 signaling pathway, a cell cycle pathway was also found to be significant, which is consistent with the p53 signaling pathway’s functional role in cell cycle progression [14] (Fig. 5).

### GeneSCF real-time mode (update mode) can detect a greater number of genes compared to other enrichment tools

GeneSCF is specifically designed to perform enrichment analysis using updated information from different functional database repositories. There are also other publicly available tools that can perform enrichment analysis on a set of gene lists. However, these tools update their functional information less frequently and thus become less reliable for performing functional enrichment analysis. To test whether the difference in the update makes any changes in the significance level of functional enrichment, we compared GeneSCF (update mode) with other publicly available tools with regularly updated databases (Table 1). We first prepared two test gene lists for a set of biological functions and pathways from the source databases GO (biological process) and KEGG. These two gene lists, A and B, were used to know the extent of difference in the number of genes detected by functional enrichment tools. List-A contains 570 genes implicated in DNA repair (GO:0006281) and Chromatin organization (GO:0006325) was obtained from GO database ‘http://geneontology.org/gene-associations/gene_association.goa_human.gz’ (Release 3/16/2016). List-B with 259 genes, represents Cell Cycle (hsa04110) and Apoptosis (hsa04210), obtained from KEGG API (Release 77.1). These two gene lists were used to validate enrichment tools to test GO and different pathway databases (KEGG and Reactome).

**Table 1:** Comparison of GeneSCF features with other functional enrichment tools

	Input type	Database update	Functional Database support	Multiple organism support	Limitations for input genes	Statistics
GeneSCF v1.1	List of genes	Real-time	KEGG, GO, Reactome and NCG	Yes	No upper or lower limit	FE test ^a^ & FDR ^b^
GeneSCF v1.0	List of genes	Manual (preparation needed)	KEGG, GO, Reactome and NCG	No (*Homo sapiens*)	No upper or lower limit	FE test & FDR
GOrilla	List of genes	Weekly auto-update	GO	Yes	No upper limit but has lower limit	FE test & FDR
DAVID 6.7	List of genes	Last known update 2010)	KEGG, GO, Reactome and Biocarta	Yes	Upper limit 3000 and no lower limit	FE test & FDR

^a^Fisher’s Exact test

^b^False Discovery Rate based methods for multiple testing correction

**Fig. 6:**
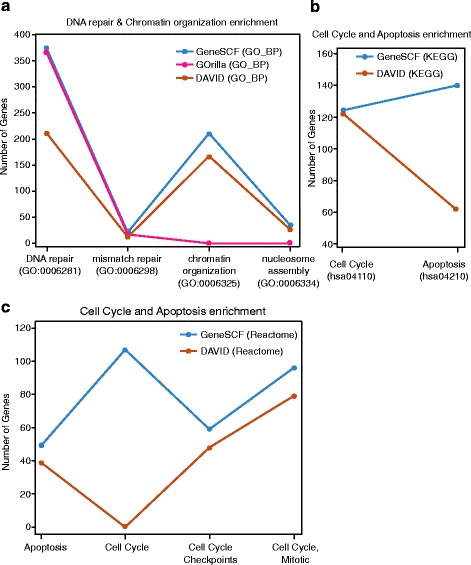
Comparison of the GeneSCF update mode with other frequently updated enrichment tools. **a** The graph shows the genes related to gene GO biological processes (GO_BP), DNA repair and chromatin organization detected by individual enrichment tools (GeneSCF, GOrilla and DAVID 6.7). The x-axis represents two biological processes considered for the analysis (GO:0006281 and GO:0006325) along with one extra biological process related to each term (GO:0006298 and GO:0006334), and the y-axis with the number of genes detected by enrichment tools. **b** The graph shows the genes related to KEGG pathways, Cell Cycle and Apoptosis detected by GeneSCF and DAVID using KEGG as a reference database. The x-axis represents pathways considered for the analysis and the y-axis with number of genes detected by enrichment tools. **c** The graph shows the genes related to KEGG pathways, Cell Cycle and Apoptosis detected by GeneSCF and DAVID using Reactome as a reference database. The x-axis represents two pathways considered for the analysis (Apoptosis and Cell Cycle) along with one extra biological process related to each term (Cell Cycle Checkpoints and Cell Cycle, Mitotic), and the y-axis with number of genes detected by enrichment tools

For comparison, we have considered enrichment tools that are closely related to the methodology of GeneSCF. In simple terms, the tools that accept gene lists as input and perform enrichment using Fisher’s Exact test statistics based on overlaps. The functional enrichment analysis was performed with gene list-A using GeneSCF and publicly available enrichment tools such as DAVID 6.7 [15], GOrilla [16] and with Gene Ontology biological process (GO_BP) as a reference database (Fig. 6a). Ideally the biological process such as DNA repair and Chromatin organization should be enriched with gene list-A. GeneSCF and DAVID showed enrichment of genes in these two processes but GOrilla could not detect one of the processes (Chromatin organization). The difference in number of genes between these tools clearly proves the lack of frequent update from DAVID and GOrilla (Fig. 6a and Additional file 10). Unlike GeneSCF, DAVID and GOrilla does not update the functional information in real-time while performing enrichment analysis, therefore there were fewer genes detected compared to GeneSCF. Since GOrilla does not support databases other than GO, further comparison of GeneSCF was made only with DAVID using list-B. Both GeneSCF and DAVID showed enrichment of Cell Cycle and Apoptosis with B-gene list using KEGG as a reference database. However, DAVID covered only 50 % (61 genes) of the genes that were covered by GeneSCF (123 genes) for the Apoptosis pathway (hsa04210) (Fig. 6b and Additional file 11), indicating that the GeneSCF update mode provides better functional enrichment information. Similarly, when gene list-B was used against the Reactome database, GeneSCF performed well compared to DAVID 6.7 in detecting Cell Cycle as a functional enrichment term (Fig. 6c and Additional file 12). This indicates that DAVID has a poorly updated Cell Cycle term in the Reactome reference database (Table 1).

The above comparisons imply that functional enrichment tools need to be updated on a regular basis to make enrichment analysis more reliable. Since GeneSCF performs analysis by directly using the information from corresponding repositories, users need not depend on the tools to update the functional information on a regular basis. In GeneSCF, the task of updating the databases is handled by users in a simpler and more flexible way.

### Advantages of using GeneSCF over other web or application interface dependent tools

Since GeneSCF is a command-line tool, the users can perform enrichment analysis using any number of gene lists on multiple organisms simultaneously by using simple bash script. Thus GeneSCF can save ample amount of time for the users compared to the users who use web interface (or application) based enrichment tools like DAVID, GOrilla, FunRich [17], Enrichr [18], etc., where manual intervention is needed to upload individual gene lists to perform the analysis and also to retrieve or save the results. Therefore GeneSCF will be extensively useful for computational biologist to integrate functional annotation or enrichment tool with their next-generation data analysis pipeline (example, differential expression analysis followed by functional significance of enriched genes). Most importantly, it is also an reliable tool compared to commonly used freely available tools because of its real-time feature. GeneSCF can be more useful when users need to perform enrichment analysis with multiple gene lists obtained by analyzing larger and multiple datasets such as The Cancer Genome Atlas (TCGA) [19], CCLE and ENCODE.

## Conclusions

A crucial part of functional enrichment analysis is using an updated reference functional database. Very few enrichment tools update their database on a regular basis and some of them require manual intervention. GeneSCF overcomes the problems associated with the coverage of updated information from functional database repositories. It is more flexible in selecting the appropriate database for multiple organisms and fetches up-to-date information from remote repositories in real-time. GeneSCF users do not have to prepare their own reference database for functional enrichment analysis. Since this tool is not a web-based tool, it does not have limitations on the number of input genes. This tool can be easily integrated into other available pipelines for transcriptome, genome-wide or other studies without need for coding or installation procedures. This reduces the time and effort for computational biologists to follow the instructions in formatting and preparing the reference database according to tool specifications. However, the major limitation of GeneSCF is that the current version works only in a Linux environment and supports two formats of input gene lists (Entrez Geneid, gid and Gene symbols, sym). Currently this tool has been tested on Linux systems and can be further extended to other Unix based operating systems like OSX. The future versions of GeneSCF can be improved to accommodate multiple input gene list formats. Since GeneSCF is easily adaptable to new functional databases and organisms, it can be extended to support a greater number of functional and cancer database repositories.

## Additional files

Additional file 1: The list of organisms supported by GeneSCF for KEGG pathway database. (XLS 542 kb) Additional file 2: The list of organisms supported by GeneSCF for Gene ontology functional database. (XLS 9 kb) Additional file 3: The list of CLL associated differentially expressed genes used for case study 1 and the list of p53 bound genes in HCT116 cell line treated with DNA-damage-inducing drug at two different time points (0 and 12 h) used for case study 2. (XLS 273 kb) Additional file 4: Cancer type enrichment results from GeneSCF for CLL associated differentially expressed genes (Case study 1) predicted using NCG as reference database. (XLS 17 kb) Additional file 5: Pathway enrichment results from GeneSCF for CLL associated differentially expressed genes (Case study 1) predicted using KEGG as reference database. (XLS 230 kb) Additional file 6: Pathway enrichment results from GeneSCF for p53 bound genes at 0 h (Case study 2) predicted by GeneSCF using KEGG as a reference database. (XLS 32 kb) Additional file 7: Pathway enrichment results from GeneSCF for p53 bound genes at 12 h (Case study 2) predicted by GeneSCF using KEGG as a reference database. (XLS 55 kb) Additional file 8: Functional enrichment results from GeneSCF for p53 bound genes at 0 h (Case study 2) predicted by GeneSCF using Gene ontology, Molecular Function (GO_MF) as a reference database. (XLS 42 kb) Additional file 9: Functional enrichment results from GeneSCF for p53 bound genes at 12 h (Case study 2) predicted by GeneSCF using Gene ontology, Molecular Function (GO_MF) as a reference database. (XLS 97 kb) Additional file 10: Enrichment analysis results for list-A genes from GeneSCF, DAVID and Gorilla using Gene Ontology, Biological Process (GO_BP) as a reference database. (XLS 1589 kb) Additional file 11: Enrichment analysis results for list-B genes from GeneSCF and DAVID 6.7 using KEGG as a reference database. (XLS 112 kb) Additional file 12: Enrichment analysis results for list-B genes from GeneSCF and DAVID 6.7 using Reactome as a reference database. (XLS 233 kb) Additional file 13: GeneSCF v1.1 tool, documentation and instructions to run test dataset (case study 1 and 2) used in this study. (GZ 4447 kb)

## Abbreviations

GeneSCF: Gene Set Clustering based on Functional annotations
KEGG: Kyoto Encyclopedia of Genes and Genomes
GO: Gene Ontology
NCG: Network of Cancer Genes
HGNC: HUGO Gene Nomenclature Committee
HUGO: Human Genome Organisation
API: Application Programming Interface
HTTP: Hypertext Transfer Protocol
TCGA: The Cancer Genome Atlas
CCLE: Cancer Cell Line Encyclopedia
ENCODE: Encyclopedia of DNA Elements

## References

[1] Kanehisa M, Sato Y, Kawashima M, Furumichi M, Tanabe M (2016). KEGG as a reference resource for gene and protein annotation. Nucleic Acids Res.

[2] Ashburner M (2000). Gene ontology: tool for the unification of biology. The Gene Ontology Consortium. Nat Genet.

[3] Croft D (2014). The Reactome pathway knowledgebase. Nucleic Acids Res.

[4] Maglott D, Ostell J, Pruitt KD, Tatusova T (2005). Entrez Gene: gene-centered information at NCBI. Nucleic Acids Res.

[5] An O (2014). NCG 4.0: the network of cancer genes in the era of massive mutational screenings of cancer genomes. Database (Oxford).

[6] Gene Ontology Consortium (2015). Gene Ontology Consortium: going forward. Nucleic Acids Res.

[7] da Huang W, Sherman BT, Lempicki RA (2009). Bioinformatics enrichment tools: paths toward the comprehensive functional analysis of large gene lists. Nucleic Acids Res.

[8] Wickham H (2009). ggplot2: Elegant Graphics for Data Analysis.

[9] Mondal T (2015). MEG3 long noncoding RNA regulates the TGF-beta pathway genes through formation of RNA-DNA triplex structures. Nat Commun.

[10] Ferreira PG (2014). Transcriptome characterization by RNA sequencing identifies a major molecular and clinical subdivision in chronic lymphocytic leukemia. Genome Res.

[11] Woyach JA, Johnson AJ, Byrd JC (2012). The B-cell receptor signaling pathway as a therapeutic target in CLL. Blood.

[12] Cuni S (2004). A sustained activation of PI3K/NF-kappaB pathway is critical for the survival of chronic lymphocytic leukemia B cells. Leukemia.

[13] Sanchez Y (2014). Genome-wide analysis of the human p53 transcriptional network unveils a lncRNA tumour suppressor signature. Nat Commun.

[14] Schwartz D, Rotter V (1998). p53-dependent cell cycle control: response to genotoxic stress. Semin Cancer Biol.

[15] da Huang W, Sherman BT, Lempicki RA (2009). Systematic and integrative analysis of large gene lists using DAVID bioinformatics resources. Nat Protoc.

[16] Eden E, Navon R, Steinfeld I, Lipson D, Yakhini Z (2009). GOrilla: a tool for discovery and visualization of enriched GO terms in ranked gene lists. BMC Bioinformatics.

[17] Pathan M (2015). FunRich: An open access standalone functional enrichment and interaction network analysis tool. Proteomics.

[18] Kuleshov MV (2016). Enrichr: a comprehensive gene set enrichment analysis web server 2016 update. Nucleic Acids Res.

[19] Aran D, Sirota M, Butte AJ (2015). Systematic pan-cancer analysis of tumour purity. Nat Commun.

