# Supplementary material for: GeneSCF: a real-time based functional enrichment tool with support for multiple organisms
Source: BMC Bioinformatics. 2016 Sep 13;17(1):365. doi: 10.1186/s12859-016-1250-z (PMC5020511; doi:10.1186/s12859-016-1250-z)
Supplement: Additional file 13: — GeneSCF v1.1 tool, documentation and instructions to run test dataset (case study 1 and 2) used in this study. (GZ 4447 kb) [file 12859_2016_1250_MOESM13_ESM.gz › geneSCF-master-source-v1.1-p1/GeneSCF-Documentation_v1.1.pdf]

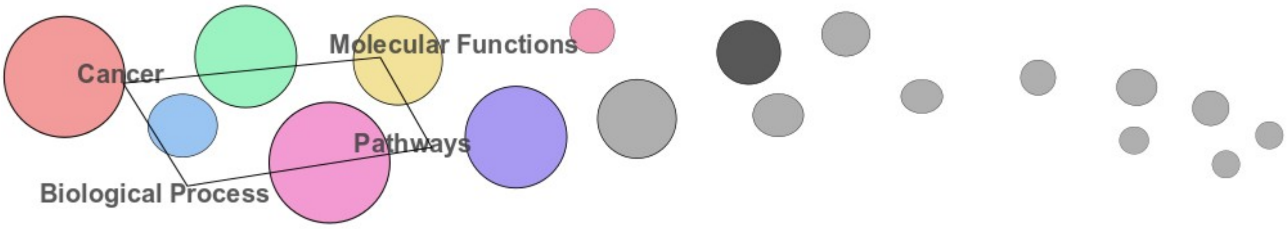

Documentation

GeneSCF v1.1: Gene Set Clustering based on Functional annotation

Santhilal Subhash

Last revised 20 January 2016

UNIVERSITY OF GOTHENBURG
SAHLGRENSKA ACADEMY
INSTITUTE OF BIOMEDICINE
Kanduri LAB
Institute of Biomedicine | University of Gothneburg | Sahlgrenska Academy
Exploring the field of Non-coding RNAs

Contents

1. Overview of GeneSCF....3
    1.1 Gene Set Clustering based on Functional annotation....3
        1.1.1 Gene Ontology....3
        1.1.2 Pathway analysis....3
        1.1.3 Cancer enrichment....3
2. Installation and prerequisites....4
    2.1 Installation....4
    2.2 System requirements....4
    2.3 Test basic UNIX commands....4
3. Detailed GeneSCF usage....5
    3.1 General usage....5
    3.2 Other modules....6
    3.3 Organisms and codes....6
4. Running test datasets (Tutorial)....7
    4.1 p53 bound genes....7
        4.1.1 Gene Ontology run....7
        4.1.2 KEGG run....8
    4.2 CLL differentially expressed genes (DEGs)....8
        4.2.1 NCG run (cancer enrichment)....8
        4.2.2 KEGG run....8
    4.3 Implementing *prepare_database* module....9
        4.3.1 Method 1 (prior database preparation)....9
        4.3.2 Method 2 (Use real-time database)....9
5. Interpreting GeneSCF output....10
    5.1 Description of output....10
    5.2 Visualizing the results....10
    5.3 Statistical methodology....11
Bibliography....12

Chapter 1

1. Overview of GeneSCF

Gene Set Clustering based on Functional annotation (GeneSCF) is a command line tool uses database from different sources KEGG, REACTOME, Gene Ontology and Network of Cancer Genes 4.0 (NCG) to find the enrichment of set of user provided gene list or target genes.

1.1 Gene Set Clustering based on Functional annotation

The statistical ranking used in this tools is an simple overlap significance Fisher's exact test employed with other multiple testing correction methods. Genes from the user supplied list were clustered using number of genes shared between the list of input genes and the genes in the biological functions from the database. The default database in GeneSCF contains functional information for Homo sapiens (Human) from KEGG, Reactome, Gene Ontology and NCG, it can also be used for more species or organisms up to 4,000 using *prepare_database* module in GeneSCF .

1.1.1 Gene Ontology

Gene ontology analysis are performed using GO database which has three sub-categories such as Biological Process, Cellular Component and Molecular Function. This tool can rank the biological functions from GO by integrating all three categories or individually based on users interest (**See section 1.3**). GO supports more than 30 organisms.

1.1.2 Pathway analysis

Pathways are used from KEGG database and could be ranked using users set of genes and it supports more than 4,000 species. Reactome is limited for organisms, it only supports the database from Homo sapiens.

1.1.3 Cancer enrichment

For cancer enrichment analysis, the database containing 2,000 genes with 66 different cancer types are used from Network of Cancer Genes 4.0 (NCG) database. NCG supports only genes from Homo sapiens.

Chapter 2

2. Installation and prerequisites

2.1 Installation

No Installation required. Please follow three simple steps below,

1. download the tool from http://genescf.kandurilab.org/downloads.php

2. Extract using TAR (tar -zxvf geneSCF-master-vx.x.tar.gz) and

3. Execute geneSCF.

2.2 System requirements

Component	Minimum requirement/Information
CPU	512 MB (1 GB recommended)
System	Linux
PERL	>=3.0 (Runs without any problem)
R-cran (Optional)	>=3.0, Only if needed addition plots
ggplot2 R-package (Optional)	Only if needed addition plots

2.3 Test basic UNIX commands

Before starting the analysis, please test these basic UNIX supportive commands for GeneSCF on the terminal.

Commands: awk, cat, gzip, wget, rm, mkdir, sort, date, sed, paste, join, grep, curl, echo, unzip, tar.

Chapter 3

3. Detailed GeneSCF usage

3.1 General usage

./geneSCF -m=[update|normal] -i=[INPATH/INPUT_FILE] -t=[gid|sym] -o=[OUTPUT_PATH/OUTPUT_FOLDER/] -db=[GO_all|GO_BP|GO_MF|GO_CC|KEGG|REACTOME] -p=[yes|no] -bg=[#TotalGenes] -org=[see,org_codes_help]

**Arguments / Parameters**	**Options / Description**
[-m= | --mode=]	For normal mode use 'normal' and for update mode use 'update' without quotes.
[-i= | --infile=]	Input file contains list of Entrez GeneIDs or OFFICIAL GENE SYMBOLS. The genes must be new lines seperated (One gene per line).
[-t= | --gtype=]	Type of input in the provided list either Entrez GeneIDs 'gid' or OFFICIAL GENE SYMBOLS 'sym' (Without quotes, Example for human 'sym' => HUGO gene symbols).
[-db= | --database=]	Database to find gene enrichment which is either geneontology 'GO_all' or geneontology-biological_process 'GO_BP' or geneontology-molecular_function 'GO_MF' or geneontology-cellular_components 'GO_CC' or kegg 'KEGG' or reactome 'REACTOME' or Network of Cancer Genes 'NCG' (Without quotes).
[-o= | --outpath=]	Existing directory to save output file. The output will be with saved in the provided location as {INPUT_FILE_NAME}_{database}_functional_classification.tsv (tab-seperated file).
[-bg= | --background=]	Total background genes to consider (Example : ~20,000 for human).
[-org= | --organism=]	Please see organism codes(For human in KEGG

Arguments / Parameters	Options / Description
	->'hsa' in Geneontology → 'goa_human'). For REACTOME and NCG use 'Hs' (human).
[-p= | --plot=]	For additional graphical output use 'yes' or 'no'.This requires R version > 3.0 and 'ggplot2' R package to be pre-installed on the system.
[-h | --help]	For displaying this help page.

3.2 Other modules

./prepare_database -db=[GO_all|GO_BP|GO_MF|GO_CC|KEGG|REACTOME|NCG]
-org=[see,org_codes_help]

Arguments / Parameters	Options / Description
[-db= | --database=]	Options:[GO_all|GO_BP|GO_MF|GO_CC|KEGG|REACTOME]
[-org= | --organism=]	Options:[see,org_codes_help]

3.3 Organisms and codes

**Multi-organism supported databases**	**organism codes**
KEGG	*GeneSCF-master-vx-x/org_codes_help* **OR** http://rest.kegg.jp/list/organism (Column 2)
GO (GO_BP,GO_CC,GO_MF,GO_all)	*GeneSCF-master-vx-x/org_codes_help* **OR** http://www.geneontology.org/gene-associations/go_annotation_metadata.all.json ("id:")

**Single-organism supported databases**	**organism code (Homo sapiens or Human)**
REACTOME or NCG	Hs

Chapter 4

4. Running test datasets (Tutorial)

GeneSCF comes with test datasets from two studies conducted in organism Human. One with list differentially expressed genes between healthy individuals and Chronic Lymphocytic Leukemia (CLL) patients. The second study with two gene lists containing p53 bound genes from 0 hours and 12 hours respectively (For detailed information about datasets refer GeneSCF article).

In this tutorial the task is to implement all type of databases from GeneSCF on these three different gene lists.

4.1 p53 bound genes

4.1.1 Gene Ontology run

>>>./geneSCF -m=update -i=test/H0.list -o=test/output/ -t=sym -db=GO_MF -bg=20000 --plot=yes -org=goa_human

The above run will search the gene list against Gene Ontology database (-db) and its sub-category Molecular Function (GO_MF) to find the enrichment. In this example, number of background genes (-bg) is 20,000 and organism (-org) Human goa_human (more organism codes refer 'org_codes_help' folder in GeneSCF). Selection of --mode parameter will determines whether GeneSCF should do enrichment run using stored database for Human on GeneSCF (-m=normal) or to use real-time database by directly connecting to GO_MF (-m=update). If 'update' mode used for already stored database on GeneSCF, the old GO database for human will be updated automatically to the recent information from GO. Since the list of input genes are from HUGO gene symbols the parameter for --type or -t is represented as 'sym'.

>>>./geneSCF -m=normal -i=test/H12.list -o=test/output/ -t=sym -db=GO_MF -bg=20000 --plot=yes -org=goa_human

This command is similar to the previous run except that we are using different list of genes and also importantly we are running in 'normal' mode. The reason for using 'normal' mode on second run is because once the 'update' mode is used for particular organism and database will be stored in GeneSCF. In this case the GO database for Human would have updated on first run and there is no need to use 'update' mode on consecutive runs for same organism and same database to reduce GeneSCF analysis time.

4.1.2 KEGG run

Similarly use the same dataset to see enriched pathways from KEGG for Human (hsa). For more organism codes refer 'org_codes_help' folder in GeneSCF.

>>>./geneSCF -m=update -i=test/H0.list -o=test/output/ -t=sym -db=KEGG -bg=20000 --plot=yes -org=hsa

>>>./geneSCF -m=normal -i=test/H12.list -o=test/output/ -t=sym -db=KEGG -bg=20000 --plot=yes -org=hsa

4.2 CLL differentially expressed genes (DEGs)

4.2.1 NCG run (cancer enrichment)

>>>./geneSCF -m=normal -i=test/TumorNormal_fc2.list -o=test/output/ -t=sym -db=NCG -bg=20000 --plot=yes -org=Hs

Above run searched the CLL DEGs against Network of Cancer Genes (NCG) database to find whether the obtained genes has any nrich ment in different cancer types. NCG does not support 'update' mode.

4.2.2 KEGG run

>>>./geneSCF -m=normal -i=test/TumorNormal_fc2.list -o=test/output/ -t=sym -db=KEGG -bg=20000 --plot=yes -org=hsa

Above run searches the CLL DEGs against KEGG pathway database to find molecular pathways which are affected in CLL. The 'normal' mode is used because of the previous run from first dataset (**section 3.1.2**).

4.3 Implementing prepare_database module

This module prepares the required database prior to enrichment run. For example, the enrichment analysis in GeneSCF can be done in two different ways

4.3.1 Method 1 (prior database preparation)

Modified example from **section 3.1.1**,

>>> ./prepare_database -db=GO_MF -org=goa_human

>>>./geneSCF -m=normal -i=test/H0.list -o=test/output/ -t=sym -db=GO_MF -bg=20000 --plot=yes -org=goa_human

In this method user will first prepare the required database (GO_MF) for an organism (goa_human) and then run the enrichment in 'normal' mode. This method will be useful for storing multiple databases for multiple organisms locally on GeneSCF for future enrichment analysis. And this can reduce time by using 'normal' mode on consecutive batch jobs on multiple databases.

4.3.2 Method 2 (Use real-time database)

Example from **section 3.1.1**,

>>>./geneSCF -m=update -i=test/H0.list -o=test/output/ -t=sym -db=GO_MF -bg=20000 --plot=yes -org=goa_human

In this method the GeneSCF uses in-built '*prepare_database*' module to connect remote repository and utilize updated database for enrichment analysis (when specified -m='update'). This is one step run for **Method 1**, this is useful when user want to use updated database on each run.

Chapter 5

5. Interpreting GeneSCF output

The output from GeneSCF contains simple tab-separated (.tsv) file containing enriched functions and corresponding group of genes with some statistical tests. The file contains 12 columns and it is explained in detail below.

5.1 Description of output

# Column	Column names	Column description
Column 1	Genes	Matched user genes list for corresponding function
Column 2	Process~name	Name of matching function / Cancer type
Column 3	num_of_genes	Number of hits in the functional database from user gene list
Column 4	gene_group	Total number of genes involved in corresponding functions
Column 5	Percentage%	Percentage of functional genes covered by user gene list
Column 6	P-value	Probability of enrichment using Fisher's exact test
Column 7-12	False Discovery Rate method	Multiple testing correction methods

5.2 Visualizing the results

When specified –plot parameter, GeneSCF plots top 20 enriched functions ranked using log-transformed P-value. This requires installation of R and 'ggplot2' package (S**ee section 2.2**). The bubble plot below shows the enrichment of CLL DEGs (**section 3.2.1**) in Leukemia and Non-hodgkin lymphoma with p-value < 0.05 significance level. The size of bubble represents the percentage of functional genes (in this case Cancer genes) covered (Column 5 from the TSV file).

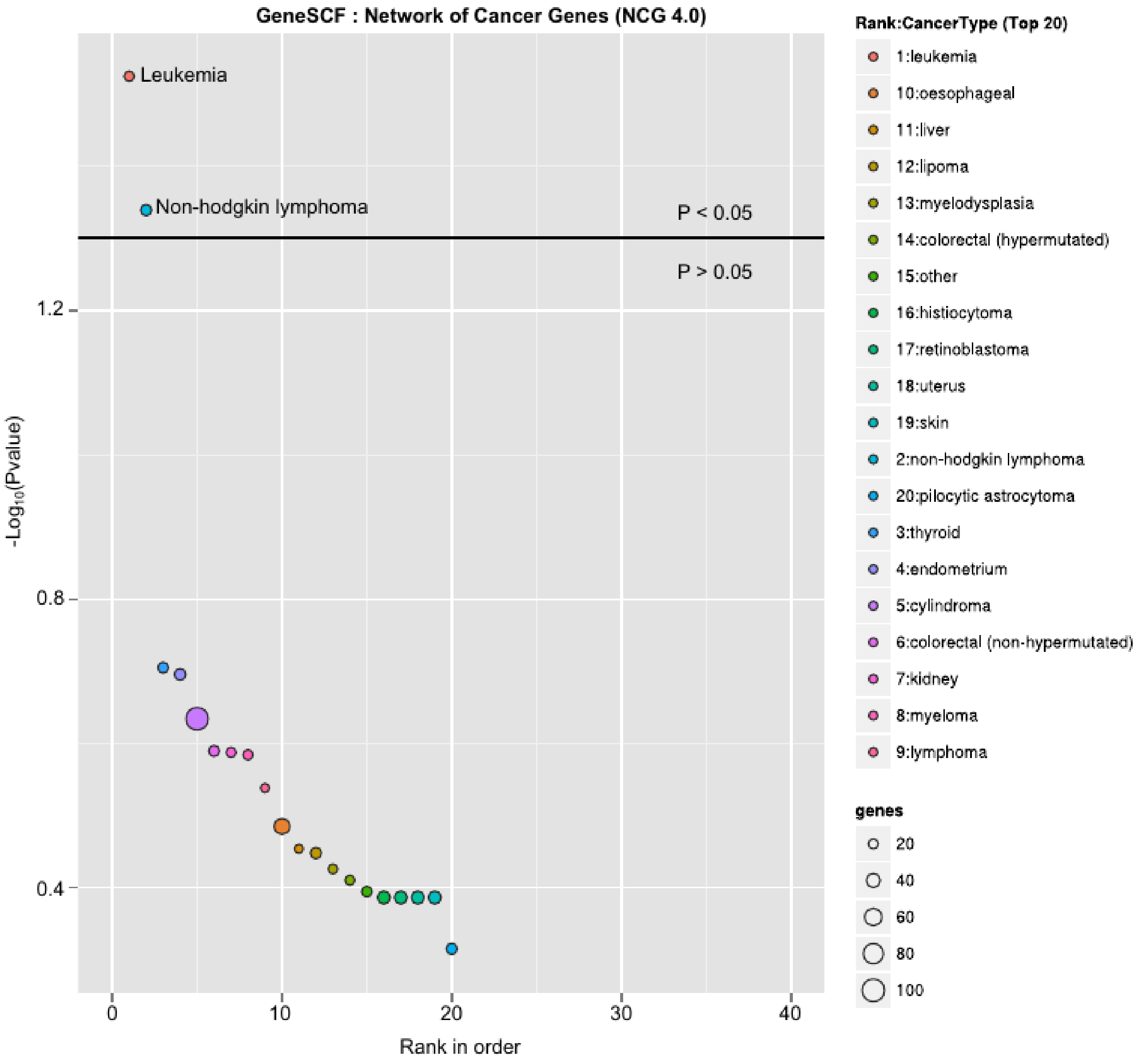

5.3 Statistical methodology

GeneSCF uses commonly used overlap statistics of Fisher's exact test and for multiple testing corrections it uses different methods. Please refer GeneSCF article for detailed methodology.

Bibliography

1. Kanehisa, M., Sato, Y., Kawashima, M., Furumichi, M. & Tanabe, M. KEGG as a reference resource for gene and protein annotation. *Nucleic Acids Res.* gkv1070– (2015). doi:10.1093/nar/gkv1070

2. Ashburner, M. *et al.* Gene ontology: tool for the unification of biology. The Gene Ontology Consortium. *Nat. Genet.* **25,** 25–9 (2000).

3. Croft, D. *et al.* The Reactome pathway knowledgebase. *Nucleic Acids Res.* **42,** D472–7 (2014).

4. Maglott, D., Ostell, J., Pruitt, K. D. & Tatusova, T. Entrez Gene: gene-centered information at NCBI. *Nucleic Acids Res.* **33,** D54–8 (2005).

5. An, O. *et al.* NCG 4.0: the network of cancer genes in the era of massive mutational screenings of cancer genomes. *Database (Oxford).* **2014,** bau015 (2014).

6. Huang, D. W., Sherman, B. T. & Lempicki, R. A. Bioinformatics enrichment tools: paths toward the comprehensive functional analysis of large gene lists. *Nucleic Acids Res.* **37,** 1–13 (2009).

7. Wickham, H. *ggplot2: Elegant Graphics for Data Analysis.* (Springer-Verlag New York, 2009). at <http://had.co.nz/ggplot2/book>

8. Ferreira, P. G. *et al.* Transcriptome characterization by RNA sequencing identifies a major molecular and clinical subdivision in chronic lymphocytic leukemia. *Genome Res.* **24,** 212–26 (2014).

9. Sánchez, Y. *et al.* Genome-wide analysis of the human p53 transcriptional network unveils a lncRNA tumour suppressor signature. *Nat. Commun.* **5,** 5812 (2014).
